# Automated resolution of the spiral torsion spring inverse design problem

**DOI:** 10.1038/s41598-024-53404-6

**Published:** 2024-02-05

**Authors:** Alejandro Silva, Gonzalo López-Navarrete, Carlos García-Martos, Juan Manuel Muñoz-Guijosa

**Affiliations:** 1 Department of Applied Mathematics, Escuela Técnica Superior de Ingenieros Industriales, Universidad Politécnica de Madrid, Calle de José Gutiérrez Abascal 2, 28006 Madrid, Spain; 2 Department of Mechanical Engineering, Escuela Técnica Superior de Ingenieros Industriales, Universidad Politécnica de Madrid, Calle de José Gutiérrez Abascal 2, 28006 Madrid, Spain

**Keywords:** Mechanical engineering, Applied mathematics

## Abstract

Many mechanical applications take advantage of spiral torsion springs due to their robustness, compactness, and simplicity. Brand-new manufacturing methods allow to create spiral springs with unconventional geometries and materials that suit a wider range of uses demanding either linearity or nonlinearity. Designing a spiral torsion spring with a nonlinear desired torque curve may be a great challenge, due to their many degrees-of-freedom (length, width, thickness, arbor, and barrel diameters, etc.) and the complexity of the geometrical and mechanical requirements to ensure their manufacturability, system compatibility, operation safety and reliability; and the solution is never unique. This manuscript proposes and validates an innovative methodology for the resolution of this inverse design problem based on the application of a nonlinear restrained global optimization algorithm. This algorithm is adjusted to converge, out of the infinity of designs that match the desired torque curve and hold all the functional and manufacturing constraints, to a design solution that minimizes strip mass. The methodology is built on a formulation for the calculation of the torque curve of a generalized spiral spring, with or without coiling and with any along-the-length cross-section, already published by the authors.

## Introduction

Spiral torsion springs are machine elements composed of a spiral strip attached to an external housing or barrel and to an arbor or inner shaft. Spiral springs can store great amounts of energy in a relatively small space due to strip bending under arbor rotation, being the stored energy a function of the bending curvatures along-the-strip length. Thanks to their high robustness and simplicity and their low cost, they are found in an ever increasing number of applications such as actuation and deployment in space robotics^1–3^, legged robots^4^, wearable robotics^5^, in micro and macro energy harvesters^6,7^, double-mass flywheels in automobile applications^8^, ballistics^9^, vibration isolation systems^10^, switchgears^11^, medical prostheses^12^, high voltage cable stressioners in railway lines, electric staplers or nail guns.

If their free strip length varies as the arbor rotates, spiral torsion springs can exhibit variable torsional stiffness^13^. To achieve this, the strip must coil and uncoil around the barrel and/or the arbor. The coiling of strip length that was previously uncoiled decreases the free coil length and increases stiffness. On the contrary, the uncoiling of strip length previously coiled increases the free coil length available, decreasing stiffness. This feature has been very useful in applications such as horology, counterbalances, tool balancers, cable reels, door actuation systems or safety belt retractors; and nowadays it has been applied for highly efficient servo-actuated balancing mechanisms^14^. An even sharper nonlinearity could be attained if the arbor and barrel coiling is combined with strip-along-the-length variable bending stiffness. Traditionally, the latter was impossible to achieve due to limitations in the strip manufacturing processes, but recent technologies such as additive manufacturing and FRPs (Fibre-Reinforced Polymers: composite materials made of fibers embedded in a polymer matrix) bring the possibility of producing strips with over-the-length variable cross-section, lattice truss, variable density core and even variable material strips. With these advanced designs and constructions, new industrial applications could be found for spiral torsion springs.

Many works have concerned in the past with the properties of spiral torsion springs under very restrictive suppositions, such as the non-existence of coiling or friction-less operation, and/or simple geometries such as the Archimedean or the logarithmic spiral^15–26^. However, the proposed models were insufficient to predict the mechanics of the more complex spiral springs with variable stiffness that can be produced with those advanced manufacturing techniques. To bridge the gap between the possibility of complex spiral spring design for novel potential applications and the knowledge of the mechanical possibilities that these designs can offer, the authors of^27^ formulated the calculation of energy-angle and torque–angle curves, strip elasticae, and contact, reaction and friction forces for spiral springs, regardless of their geometry, the variability of the cross-section and the material properties with the length, and the existence or non-existence of coiling about either the arbor for the barrel. The only model hypotheses are staticity, homogeneity, monolithycity, inextensibility and no interference between spires. These assumptions are reasonable under the mechanical and industrial conditions under which most spiral springs operate. The calculation of the mechanical features of a spiral spring from its geometry and material properties is what is named the *direct problem* of spiral torsion springs.

A more ambitious and challenging task is the resolution of the *inverse design problem* of the spiral torsion spring: to find a spring design with a desired angle-torque curve and the lowest possible mass and material expenditure while holding a set of geometrical and mechanical constraints guaranteeing that the design is feasible to manufacture and to assemble, that it is a good fit to the application of interest and that it is mechanically resistant. In the recent past, the resolution of the inverse problem has found a few interesting applications in Mechanical Engineering such as the design of soft filaments^28^, structures^29^, airfoils^30^, manufacturing processes for hot steel rods^31^, injection molds^32^, and many others^33^. A few studies have been devoted in the past to optimal design of spiral springs, such as^34–36^ However, these studies considered spiral springs with very simple geometries, constant cross section, material homogeneity and no coiling (hence linear or almost linear springs).

In this manuscript, A method to solve the inverse design problem of the generalized spiral torsion spring is proposed. The basis of this method is the spiral spring model described in^27^. The methodology is based on the use of nonlinear restrained global optimization algorithms. Our work is based on a heuristic optimization algorithm known as the *genetic algorithm*^37^. It draws from the principles of biological, Darwinian evolution to find a minimum or maximum of a function subject to certain mathematical constraints. Heuristic algorithms have already been proposed for the resolution of some inverse problems in Mechanics, for example in^28,29,33^. If the population sizes and the number of generations are sufficiently large, genetic algorithms have the potential to converge to the global extremum inside the domain of feasible solutions^38^. This is an advantage when optimizing complicated functions with multiple minima.

This work begins (Section “Formulation of the inverse problem for the design of spiral springs”) with the formulation of our proposed methodology for automatic constrained design of spiral springs given a desired torque–angle curve and geometrical and material properties. Section “Results and discussion” proves the great potential of the proposed methodology for efficient design and mass reduction and describes this experimental validation of this methodology for constant cross-section springs and variable-width spring. The manuscript ends with the conclusions (Section “Conclusions”).

## Formulation of the inverse problem for the design of spiral springs

### Flow diagram of the resolution of the inverse spring design problem with the genetic algorithm

**Figure 1:**
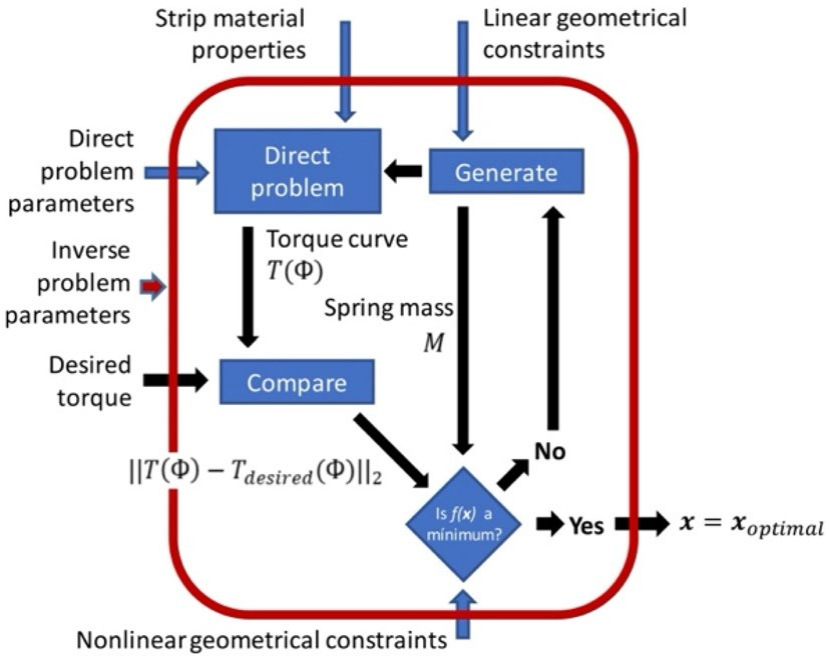
The strategy followed for the resolution of the inverse design problems of spiral springs.

Figure 1 sums up the proposed methodology for automated spiral spring design. The inverse problem algorithm has these inputs: the wanted torque-arbor angle curve: ${T}_{desired}\left(\Phi \right)$, the mechanical properties of the strip and arbor material: $E$, $\rho$, ${\tau }_{adm}$ and ${\sigma }_{adm}$; the geometrical constraints and boundaries imposed on the strip such as ${\Phi }_{B}$, ${L}_{min}$, ${L}_{max}$, ${k}_{min}$ and ${k}_{max}$; the parameters of the direct problem that determine strip discretization and the numerical calculation of $T\left(\Phi \right)$: $m$ (the number of strip nodes), $N$ (the number of steps in the calculation of $T\left(\Phi \right)$), $\Delta {\Phi }_{desired}$ (the desired minimum angular range, determined by $T\left(\Phi \right)$), the parameters that enable polynomial interpolation of the strip length-curvature function for dimensionality reduction^39^, and the settings of the numerical optimization algorithm, including ${K}_{torque/mass}$ (in Eq. 27) and ${K}_{penalty}$ (in Eq. 29). Its output is a vector $x$ containing the optimal geometrical parameters of a spring that solves this inverse problem: $L$, $k$, ${h}_{1}$, ${h}_{m}$, ${b}_{1}$, ${b}_{m}$, ${r}_{H}$ and ${r}_{S}$.

The way new candidate solutions are generated in the framework described in Fig. 1 if the current candidate solution $x$ is not good enough varies from optimization algorithm to algorithm. In this manuscript, the heuristic *genetic algorithm* (GA) has been chosen to solve the inverse problem of the generalized spiral spring. Algorithmic details on the parameters and implementation of the genetic algorithm are shown in the supplementary materials document.

### Vector of design characteristics of a spiral spring and its constraints

Proper application of a genetic algorithm to solve the inverse design problem of spiral torsion springs requires the definition of a minimal set of design parameters with a one-to-one correspondence with a feasible spiral spring. These parameters are elements of a *vector of characteristics* ${\varvec{x}}$ defining one individual of a population of feasible springs. In accordance with the direct problem assumptions in Section S1 of the supplementary material and to some geometric simplifications, we propose the following vector to characterize a spiral spring:

1 $${\varvec{x}}=\left(L,k,{h}_{1},{h}_{m},{b}_{1},{b}_{m},{r}_{H},{r}_{S}\right),$$

where $L$ is the strip length, $k$ is the pitch angle of the spiral in manufacturing state (that is, before its assembly to the barrel and arbor), ${h}_{1}$ and ${h}_{m}$ are strip thicknesses at the first and last strip nodes, ${b}_{1}$ and ${b}_{m}$ are strip widths at those very same nodes, and ${r}_{H}$ and ${r}_{S}$ are barrel and arbor radii, respectively.

For simplicity of design, we will consider only strips whose manufacturing geometry is shaped as a logarithmic spiral centered at the arbor rotation axis and with a linear variation of the thickness and width along the coil length (with rectangular cross-section) so that the clamping angle between the coil and the arbor surface will be equal to the coil shape parameter $k$. These assumptions are not a part of the original inverse-problem formulation; in fact, the proposed methodology can be applied to any torsion spring design modelable under the five direct problem hypotheses in Section S1 of the supplementary material. The assumption of a logarithmic spring with linearly varying cross-section allows us to simplify the computational implementation, constraining it to a subset of all the feasible spring designs holding the initial hypotheses.

With these presumptions, we can calculate the element widths and thicknesses at the remaining strip nodes $i=2,\text{...},m-1$ using the following expressions:

2 $${h}_{i}={h}_{1}+\frac{i-1}{m-1}\left({h}_{m}-{h}_{1}\right),$$

3 $${b}_{i}={b}_{1}+\frac{i-1}{m-1}\left({b}_{m}-{b}_{1}\right).$$

As for the nodal curvatures, they can be calculated from the coil length $L$, its pitch angle $k$ and ${r}_{S}$ as follows: let $l$ be the strip length coordinate, with $l=0$ at the attachment to the arbor, and $l=L$ at the attachment to the barrel. Since the coil is shaped as a logarithmic spiral centered at the arbor rotation axis, the dependence of the strip radius with the axial strip position can be expressed as^25^

4 $$l=\frac{\sqrt{1+{k}^{2}}}{k}\left(R\left(l\right)-{r}_{S}-\frac{{h}_{m}}{2}\right)\to R\left(l\right)={r}_{S}+\frac{{h}_{m}}{2}+\frac{kl}{\sqrt{1+{k}^{2}}}.$$

Then, the strip manufacturing curvatures can be obtained from $R\left(l\right)$ with the formula.

5 $${c}_{0}\left(l\right)=\frac{1}{R\left(l\right)\sqrt{1+{k}^{2}}}.$$

Additional geometrical constraints imposed on the spiral springs must ensure that its dimensions are appropriate for its application, that it can be properly manufactured and assembled and that it has mechanical resistance and durability. The linear constraints that the eligible springs must hold are enlisted as follows:

6 $${r}_{S}\le {r}_{H},$$

7 $${L}_{min}\le l\le {L}_{max},$$

8 $${k}_{min}\le k\le {k}_{max},$$

9 $${h}_{1,min}\le h\le {h}_{1,max},$$

10 $${h}_{m,min}\le h\le {h}_{m,max},$$

11 $${b}_{i,min}\le b\le {b}_{i,max},$$

12 $${b}_{m,min}\le b\le {b}_{m,max},$$

13 $${r}_{H,min}\le {r}_{H}\le {r}_{H,max},$$

14 $$max\left({r}_{S,min},{r}_{S,shear}\right)\le {r}_{S}\le {r}_{S,max},$$

All the former upper and lower bounds are constraints on the spring dimensions. As a side note, ${r}_{S,shear}$ is the minimum arbor radius necessary for guaranteeing its mechanical integrity when the spring is fully loaded, that is, when the spring torque is $T\left({\Phi }_{max}\right)$. For a cylindrical arbor it equals.

15 $${r}_{S,shear}=\sqrt[3]{\frac{2T\left({\Phi }_{max}\right)}{\pi {\tau }_{adm}}}.$$

where ${\tau }_{adm}$ is the shear limit characterizing the arbor material.

The nonlinear constraints enforced on the spiral springs help to ensure proper assembly and operation, mechanical resistance and that it realizes the required specifications (namely, storage energy and angular range). The whole set of nonlinear conditions is enlisted as follows:

16 $$\sqrt{{x}_{ac\text{,1}}^{2}+{y}_{ac,1}^{2}}\le {r}_{H},$$

17 $${r}_{S}\le \sqrt{{x}_{bc,m}^{2}+{y}_{bc,m}^{2}},$$

18 $$E\left({c}_{i}-{c}_{0,i}\right){h}_{i}/2\le {\sigma }_{adm},$$

19 $${E}_{desired}/{\rho }_{E}\le V,$$

20 $$\Delta {\Phi }_{desired}\le \Delta \Phi ,$$

21 $${c}_{bc\text{,2}}\le {c}_{{\Phi }_{max}\text{,2}},$$

22 $${c}_{{\Phi }_{max}\text{,2}}\le {c}_{ac\text{,2}},$$

23 $${c}_{0,2}\le {c}_{bc\text{,2}}.$$

Equation 16 and 17 ensure that the strip can be assembled between barrel and arbor. Equation 18 must hold separately in all strip nodes to maintain the strip bending stresses below the maximum admissible stress ${\sigma }_{adm}$. Equation 19 guarantees that the strip volume $V=\int b\left(l\right)h\left(l\right)dl$ is large enough to store the elastic energy required to the spring, ${E}_{desired}$, which is.

24 $${E}_{desired}=\int {T}_{desired}\left(\Phi \right)d\Phi ,$$

where ${T}_{desired}\left(\Phi \right)$ is the desired torque-arbor angle curve, and ${\rho }_{E}={\sigma }_{adm}^{2}/\left(6E\right)$ is the energy density given by the strip material. Equation 20 guarantees that the spring angular rotation range $\Delta \Phi ={\Phi }_{max}-{\Phi }_{A}$ is at least equal to the desired range, which is given by the wanted torque–angle curve, $T\left(\Phi \right)$. $\Delta \Phi$ can be approximated for the current spring under evaluation by the expression

25 $$\Delta \Phi =\int \left({c}_{ac}\left(l\right)-{c}_{0}\left(l\right)\right)dl.$$

The last nonlinear conditions, given by Eqs. 21–23, guarantee that the whole strip length contributes to the energy storage of the spring, or equivalently, that there are no idle strip sections that never uncoil from the barrel nor the arbor. ${c}_{{\Phi }_{max}}$ is defined as the strip curvature function for an arbor angle $\Phi$ equal to the desired range, ${\Phi }_{max}$.

### The objective function of the inverse design optimization problem of spiral springs

We define the scalar objective function to optimize as $f\left({\varvec{x}}\right)$. The aim is to find a minimum of this function (if possible, a global minimum) inside the feasible domain of spiral springs whose vectors of characteristics $x$ hold all the constraints (Eqs. 6–23):

26 $${f}_{1}({\varvec{x}})+{f}_{2}({\varvec{x}})+{f}_{3}({\varvec{x}}).$$

The objective function to minimize, $f\left({\varvec{x}}\right)$, is the sum of three strictly non-negative terms ${f}_{1}\left({\varvec{x}}\right)$, ${f}_{2}\left({\varvec{x}}\right)$ and ${f}_{3}\left({\varvec{x}}\right)$. These three quantities are defined as.

27 $${f}_{1}\left({\varvec{x}}\right)={K}_{torque/mass}\frac{{\Vert T\left(\Phi \right)-{T}_{desired}\left(\Phi \right)\Vert }_{2}}{{\Vert {T}_{desired}\left(\Phi \right)\Vert }_{2}},$$

28 $${f}_{2}\left({\varvec{x}}\right)=\frac{M}{{M}_{ideal}}-1,$$

29 $${f}_{3}\left({\varvec{x}}\right)={K}_{penalty}{\sum }_{i=1}^{NNC}\left[i-th\hspace{1em}nonlinear\hspace{1em}condition\hspace{1em}not\hspace{1em}holding\right],$$

where ${\Vert f\Vert }_{2}$ denotes the ${L}_{2}$ norm (or *Euclidean* norm) of a bounded function $f$: the root square of its squared image, $\int {f}^{2}$, and the Iverson notation $\left[{P}_{i}\right]$ denotes an operation that returns 1 if the i-th statement ${P}_{i}$ is true and 0 if it is false. ${f}_{1}\left({\varvec{x}}\right)$ measures the relative distance between the desired torque–angle curve ${T}_{desired}\left(\Phi \right)$ and the one of the currently evaluated spring ${\varvec{x}}$**,** $T\left(\Phi \right)$. ${f}_{2}\left({\varvec{x}}\right)$ quantifies the relative mass of the current spring ${\varvec{x}}$, $M$, with respect to its ideal mass ${M}_{ideal}$: the minimum mass required to store the elastic energy ${E}_{desired}$.

The calculation of the torque–angle curve $T\left(\Phi \right)$ requires solving the direct problem of the spiral spring with vector of characteristics ${\varvec{x}}$. The formulation of the direct problem of the spiral spring is briefly described in the supplementary materials document. For further details on the direct problem formulation and an experimental validation, we refer the reader to Muños-Guijosa et al.^27^.

The spring mass is easily calculated from its geometry:

30 $$M=\rho {\sum }_{i=1}^{m-1}{b}_{i}{h}_{i}\Delta {L}_{i},$$

and its ideal, optimal mass is given by.

31 $${M}_{ideal}=\frac{\int {T}_{desired}\left(\Phi \right)d\Phi }{{\sigma }_{adm}^{2}/\left(6E\right)}.$$

Given a material and a torque–angle curve, the ideal spring mass is a lower bound to the mass of any spiral spring with the same material and torque–angle curve, therefore ${f}_{2}\left({\varvec{x}}\right)$ is strictly nonnegative.

${f}_{3}\left({\varvec{x}}\right)$ is a penalty term that counts the total number of nonlinear constraints (Eqs. 16–23) that the spiral spring does not hold. It is a nonnegative integer whose absolute minimum is zero. The optimal spring should hold all the nonlinear constraints; hence this quantity should converge to zero during the execution of the optimization algorithm.

The parameters ${K}_{torque/mass}$ in Eq. 27 and ${K}_{penalty}$ in Eq. 29, which are fixed before the optimization, determine the relative importance of minimizing ${f}_{1}\left({\varvec{x}}\right)$, ${f}_{2}\left({\varvec{x}}\right)$ and ${f}_{3}\left({\varvec{x}}\right)$. They must be adjusted in a manner that when finding an optimal ${\varvec{x}}$, ${f}_{1}\left({\varvec{x}}\right)$ and ${f}_{3}\left({\varvec{x}}\right)$ converge to zero while ${f}_{2}\left({\varvec{x}}\right)$ converges to a minimum positive value, in a manner that the optimal spring found holds all the constraints. A compromise must be reached between mass minimization and accuracy of the torque–angle curve. The balance of this compromise is controlled by the parameter ${K}_{torque/mass}$. Too low a value may lead to very light springs with sub-optimal torque curves, while high values lead to overweight springs.

Minimizing $f\left({\varvec{x}}\right)$ (Eq. 26) under constraints in Eqs. 6–23 should yield an optimal and feasible design solution whose torque–angle curve matches a desired curve with the lowest material use. A *genetic algorithm* has been chosen to solve this optimization problem. The following section explains in detail its application for the resolution of this inverse design problem.

## Results and discussion

The methodology described in Section “Formulation of the inverse problem for the design of spiral springs” has been implemented on Matlab R2019b in an HP ProBook 450 G3 with a 64-bit Intel Core i-6200U (2.3 GHz) and 8 GB of RAM. The parameters of the genetic algorithm have been these across all executions: the population size ${N}_{ind}$ was set to 50, the maximum number of generations was set to 50, the crossover fraction was set to 0.8, the elite count was set to 13, the initial penalty value was 10, the migration fraction was 0.2, the number of generations between migrations of individuals between sub-populations was 20, the Pareto fraction was set to 0.35 and the penalty factor was set to 100. For the resolution of the direct problem, we chose $m={1216}$ strip nodes, $N={16}$ arbor angular steps for the calculation of the torque–angle curve, and 16 sample nodes equidistant along the strip. For the objective function Eq. 26, we got optimal convergence of $f\left(x\right)$ by setting ${K}_{penalty}$ to one and ${K}_{torque/mass}$ to five in all the solved optimization cases.

**Figure 2:**
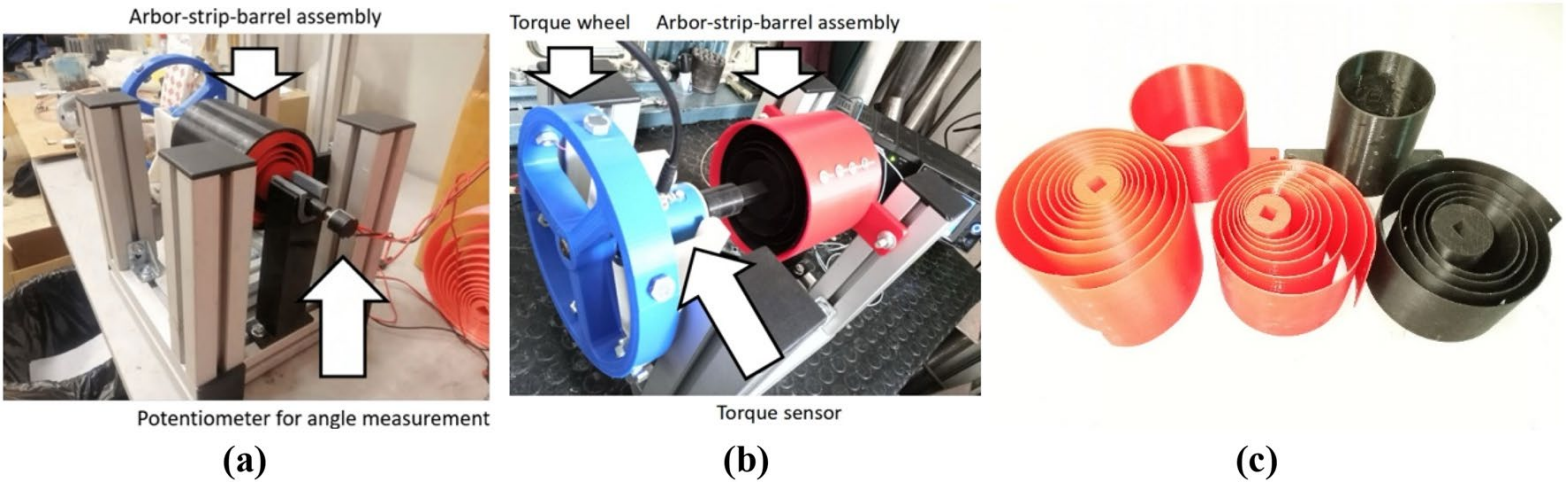
The experimental bench for the measurement of the torque–angle curve of the spiral springs. (**a**) and (**b**) Bench assembly. (**c**) The strips tested during validation.

Springs for the validation of the optimization strategy (with constant cross-section and variable width) were 3D-printed using high-density PLA with the following mechanical properties: a density $\rho$ of 1100 kg/m^3^, a Young’s modulus $E$ of 3 GPa, a tension elastic limit ${\sigma }_{adm}$ of 25 MPa, and a shear elastic limit ${\tau }_{adm}$ of 12.5 MPa. The geometrical parameters of the linear and nonlinear constraints set for the execution of the inverse problem, including boundaries of the spring features, are in Table 1. The testing bench for measuring the torque–angle curves is shown in Fig. 2 along with the validation springs manufactured. Their torque–angle curves were measured by manually spinning the blue torque wheel shown in Fig. 2a while collecting torque and angular displacement data with a potentiometer (to measure the arbor rotation angle during the tests) and a torque sensor. The combination of the two measurements on a two-dimensional plot gives the experimental torque–angle curves of each of the two spiral springs. To guarantee the consistency of the results, four loading-releasing cycles were combined during the testing of each spring design, and the measurements were averaged and interpolated to obtain the definitive experimental torque–angle curves.

**Table 1:** Geometrical parameters and boundaries of the springs for the resolution of the inverse problem.

${L}_{min}$	$2.00$ m	${L}_{max}$	${3.00}$ m
${k}_{min}$	$2\times {1}{{0}}^{-2}$ rad	${k}_{max}$	$8\times {1}{\text{0}}^{-2}$ rad
${h}_{1,min}$	$\text{1.5}\times {1}{{0}}^{-3}$ m	${h}_{1,max}$	$3\times {1}{\text{0}}^{-3}$ m
${h}_{m,min}$	$\text{1.5}\times {1}{{0}}^{-3}$ m	${h}_{m,max}$	$3\times {1}{\text{0}}^{-3}$ m
${b}_{1,min}$	$5\times {1}{{0}}^{-2}$ m	${b}_{1,max}$	$\text{1.5}\times {1}{{0}}^{-1}$ m
${b}_{m,min}$	$5\times {1}{{0}}^{-2}$ m	${b}_{m,max}$	$\text{1.5}\times {1}{{0}}^{-1}$ m
${r}_{H,min}$	$4\times {1}{{0}}^{-2}$ m	${r}_{H,max}$	$1\times {1}{{0}}^{-1}$ m
${r}_{S,min}$	$2\times {1}{{0}}^{-2}$ m	${r}_{S,max}$	$4\times {1}{{0}}^{-2}$ m

### On a nonlinear torsion spring with constant cross section

**Figure 3:**
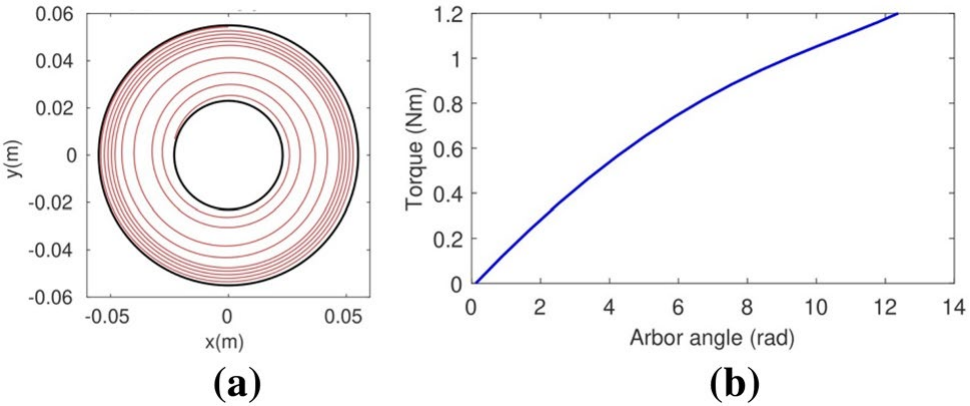
The reference spiral spring. (**a**) Its mounting position. (**b**) Its theoretical torque curve, as given by the direct problem resolution (Section S1 of the supplementary material).

The second column of Table 2 shows the dimensions, masses, and objective function’s terms values of a logarithmic spiral spring with constant cross-section used as a reference spring to validate the methodology for the resolution of the inverse design problem described in Section “Formulation of the inverse problem for the design of spiral springs”. The geometry of this reference spring was obtained by re-scaling the number four spring tested in Queener and Wood^25^ to enable its production by 3D-printing and to allow for its experimental measurement with our available instrumentation. The resolution of the inverse problem for constant cross section springs requires the introduction of two additional linear constrains into its formulation (Eq. 26): a constant width constraint (${h}_{1}={h}_{m}$) and a thickness constraint (${b}_{1}={b}_{m}$). Its mounting elastica and its theoretical torque curve, both calculated as indicated in Section S1 of the supplementary material, are shown in Fig. 3.

**Table 2:** Dimensions of the reference spiral spring and the three optimal springs (with constant cross-section, with variable width and with variable width and thickness), coil masses and values of the objective values’ terms.

	Reference	Constant cross-section	Variable width	Variable width and thickness
Width at arbor/width at barrel (cm)	15/15	6.22/6.22	5.87/6.70	5.73/6.74
Thickness at arbor/thickness at barrel (mm)	1.5/1.5	2.00/2.00	2.02/2.02	2.00/1.90
Length (m)	2.5	2.3	2.19	2.14
Arbor radius/Barrel radius (mm)	23/55	31.30/86.20	24.1/96.7	29.5/77.9
Pitch angle (rad)	0.027	0.053	0.076	0.048
Coil mass (g)	618	315	306	288
${f}_{1}\left({\varvec{x}}\right)$	0	0.220	0.400	0.655
${f}_{2}\left({\varvec{x}}\right)$	1.303	0.172	0.138	0.065
${f}_{3}\left({\varvec{x}}\right)$	0	0	0	0

**Figure 4:**
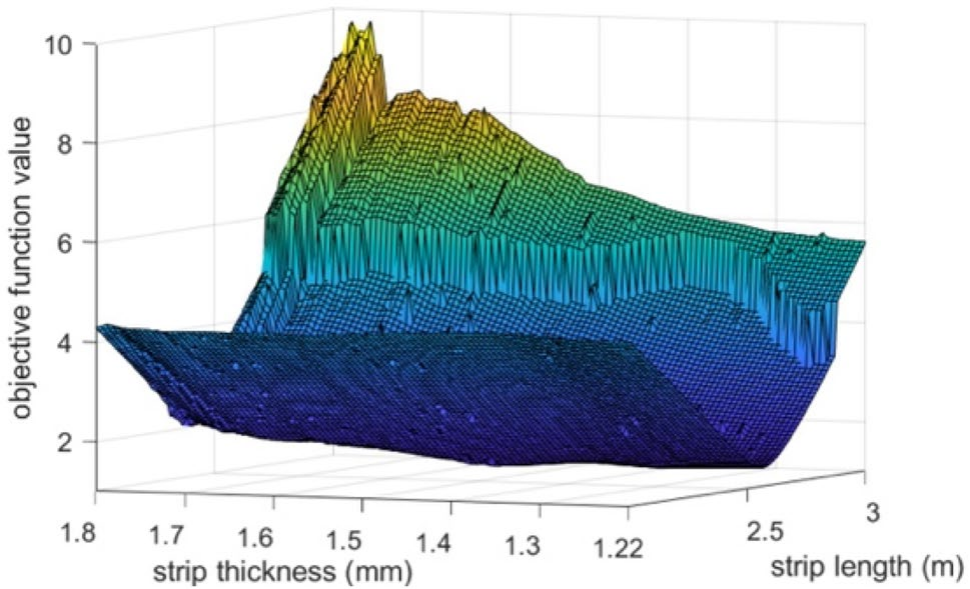
The objective function (Eq. 26) evaluated in a set of spiral springs with varying length and thickness.

An empirical analysis of the objective function (Eq. 26) based on this spiral spring has been carried out to show its deeply nonlinear nature and its non-convexity. Equation 26 was evaluated for a set of spiral springs with varying length (between 2 and 3 m) and thickness (between 1.2 and 1.8 mm) and with the remaining design parameters equal to those of the reference spring (Table 2). The resulting two-dimensional surface relating the value of the objective function to the coil length and thickness is represented in Fig. 4. The objective function shows a rift where it is expected to find a minimum in the evaluation domain. Due to the complex nature of the function, however, several local extrema along the rift are noticeable in the representation. Gradient-based deterministic optimization algorithms may converge to any of these minima, depending on the initial solution input to the algorithm. This justifies the application of heuristic methodologies for the resolution of the inverse design problem of the spiral spring.

**Figure 5:**
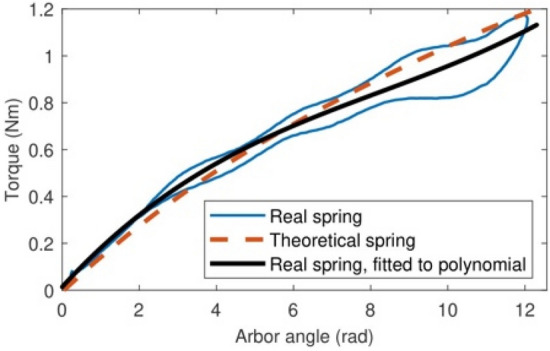
A comparison between the theoretical torque curve of the reference spiral spring (in black) and its real, measured torque curve (in blue) as approximated by the interpolating third-degree polynomial in red.

Before running the optimization of the geometry of the reference spiral spring, its theoretical torque–angle curve (Fig. 3b) was validated experimentally. Figure 5 compares the experimentally measured torque curve of the reference spring (in blue) and a third-degree interpolating polynomial that approximates it (in red) with the theoretical torque curve given by the direct problem resolution. Note how accurately the theoretical and the experimental torque–angle curves match. As a side note, the torques measured when loading the spring are slightly higher than the torques measured when releasing the spring due to friction hysteresis. Once this reference spring has been validated, we are ready to solve the inverse design problem described in Section “Formulation of the inverse problem for the design of spiral springs” to obtain designs of torsion spiral springs that attain the same torque–angle curve as in Fig. 3b with reduced material use, optimizing the strip mass.

**Figure 6:**
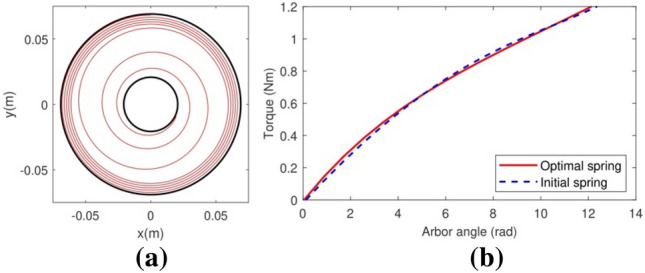
The optimal spiral spring with constant cross-section and the theoretical torque curve in Fig. 3b. (**a**) Its mounting position. (**b**) Its theoretical torque curve (in red), compared with the theoretical curve of the reference spring (dashed blue line).

**Figure 7:**
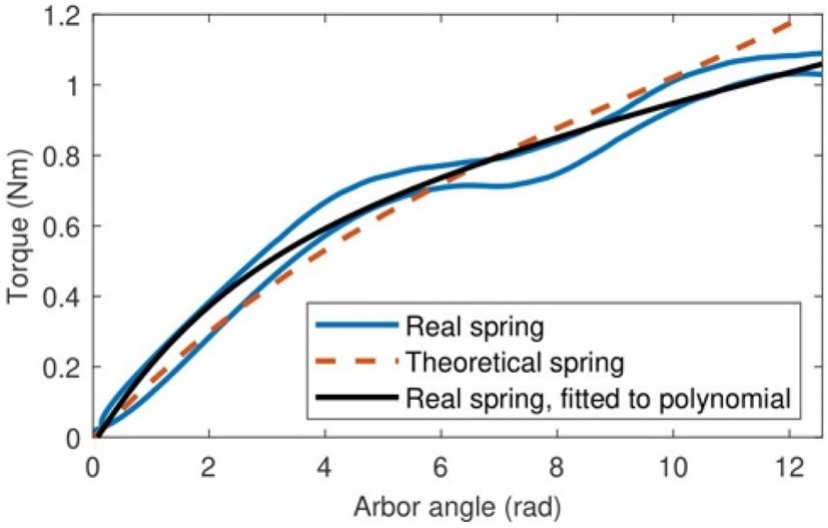
A comparison between the theoretical torque curve of the optimal spiral spring with constant cross-section (in black) and its real, measured torque curve (in blue) as approximated by the interpolating third-degree polynomial in red.

The resolution of the inverse design problem (Section “Formulation of the inverse problem for the design of spiral springs”) with the torque curve in Fig. 3b and the constraints in Table 1 output the optimal spiral spring in Fig. 6a with the dimensions shown in the third column of Table 2. This optimal spring is shorter than the reference spring and has a remarkably lower section width while their theoretical (Fig. 6b) and experimental (Fig. 7) average torque curves are like those of the reference spiral spring, as desired. Oscillations in the measured torque curves are caused by minor misalignment of the arbor with respect to the barrel and by material and geometrical inhomogeneities such as low manufacturing tolerances (about $\pm \text{0.2}$ mm in 3D printing, great enough to affect significantly the strip coiled and uncoiled lengths per unit of arbor rotation). Despite this, we argue that the results validate our methodology for the resolution of the inverse spiral-spring design problem.

### On a nonlinear torsion spring with constant thickness and variable width

Further proof of the potential and flexibility of the proposed methodology for the design of springs with unconventional geometries is given by the application of the inverse problem formulation proposed (Section “Formulation of the inverse problem for the design of spiral springs”) to the optimization of spiral springs with variable cross-section. Springs with these geometries are attained when the constant width constraint (${h}_{1}={h}_{m}$) and/or the constant thickness constraint (${b}_{1}={b}_{m}$) are removed from the formulation of the inverse design problem (Eq. 26).

**Figure 8:**
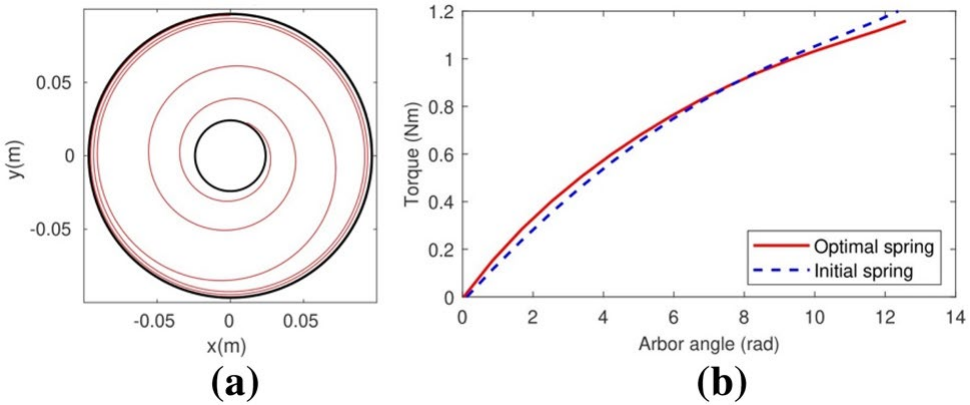
The optimal spiral spring with variable width and the theoretical torque curve in Fig. 3b. (**a**) Its mounting position. (**b**) Its theoretical torque curve (in blue), compared with the theoretical curve of the reference spring (in red).

**Figure 9:**
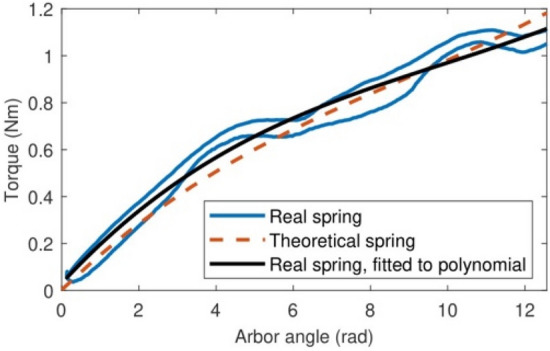
A comparison between the theoretical torque curve of the optimal spiral spring with variable width (in black) and its real, measured torque curve (in blue) as approximated by the interpolating third-degree polynomial in red.

After the removal of the constant width constraint from the inverse problem formulation, the execution of the optimization software output the spiral spring geometry shown in Fig. 8a with the dimensions shown in the fourth column of Table 2 for the torque curve in Fig. 3b. The spring strip is shorter than the two previous springs, and on average its width is the lowest of the three, therefore this spring will be even lighter than the optimal spring with constant cross-section. The theoretical torque curve of this optimal spring (Fig. 3b) is close enough to the torque curve of the reference spring (Fig. 8b). Regarding the experimental torque–angle curve of a real spring with the dimensions of the optimal variable-width spiral spring (Fig. 9), it shows the same oscillations than the optimal constant-section spring but on average it is remarkably close to the theoretical curve.

### On a nonlinear torsion spring with variable thickness and width

**Figure 10:**
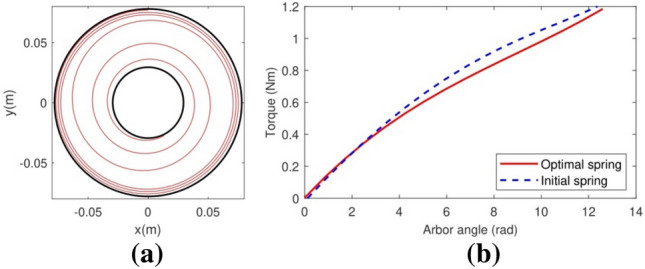
The optimal spiral spring with variable thickness and width and the theoretical torque curve in Fig. 3b. (**a**) Its mounting position. (**b**) Its theoretical torque curve (in blue), compared with the theoretical curve of the reference spring (in red).

Removing both constant thickness and width conditions and executing the optimization programs results in an optimal spiral spring with linearly variable thickness and width according to Eqs. 2 and 3 and with a torque curve that approximates the one in Fig. 3b. Figure 10a, b show, respectively, its mounting elastica and its theoretical torque–angle curve. Its dimensions are in the fifth column of Table 2. Convergence to an optimal solution is slower in the variable thickness case because of the greater number of degrees-of-freedom of the optimization problem. For this reason, the torque curve of the optimal solution obtained after 50 generations diverges notably from the desired curve. Despite being a sub-optimal spring, this is the smallest and lightest of them all, which showcases again the potential of the proposed inverse problem formulation.

## Conclusions

In this work, we demonstrate that the methodology described in Section “Formulation of the inverse problem for the design of spiral springs” for the resolution of the inverse design problem of torsion spiral springs can provide a strip design with the desired torque–angle curve. Moreover, if its parameters are properly set, the algorithm outputs an optimal spring with minimal weight within the geometrical constraints of the strip and its cross-section, while ensuring proper manufacturability and mechanical resistance. The validation of the methodology has demonstrated its potential and accuracy notwithstanding the drawbacks associated to the poor manufacturing tolerance of standard 3D printing systems based on Fused Deposition Modeling.

The accuracy of the proposed methodology for optimal automatic design of spiral springs is limited by the need to reach a trade-off between computation times, GA’s population sizes and number of generations, the precision of the calculated torque–angle curves and the complexity of the strip geometry. Future research lines should focus on finding optimization algorithms and new formulations of the inverse design problem that lead to increased accuracy, better spring design and lower computation times. In this line, we propose:

- Tuning the GA’s and the torque–angle curve calculation parameters for better precision and more optimal output spiral springs while reducing calculation times.
- Reformulating Eq. 26 as a multi-objective problem with two functions: the error of the torque–angle curve and the spring mass, under the nonlinear constraints (Eqs. 6–23), and to calculate the Pareto front of non-dominating spiral spring designs,
- Improving the spiral spring’s mathematical model and its parameters to render it more representative of real springs,
- Validating the methodology for spring coils with variable width and thickness, non-homogeneous materials, and other non-conventional designs.

### Supplementary Information

Supplementary Information.

## Data Availability

The datasets used and/or analyzed during the current study are available from the corresponding author on reasonable request.

## List of symbols

${{b}}\left({{l}}\right)$: Strip width as a function of the length coordinate
${{{b}}}_{{{i}}}$: Strip width at the i-th node
${{{b}}}_{1,{{max}}}$: Maximum admissible strip width at node one
${{{b}}}_{1,{{min}}}$: Minimum admissible strip width at node one
${{{b}}}_{{{m}},{{max}}}$: Maximum admissible strip width at the last node
${{{b}}}_{{{m}},{{min}}}$: Minimum admissible strip width at the last node
${{{c}}}_{{\Phi }_{{{max}}}{,i}}$: I-th node strip curvature at the maximum arbor rotation angle
${{{c}}}_{0}\left({{l}}\right)$: Manufacturing strip curvatures as a function of the length coordinate
${{{c}}}_{{{ac}}{,i}}$: Arbor coiling curvature at the i-th node
${{{c}}}_{{{ac}}}\left({{l}}\right)$: Arbor coiling curvature as a function of the length coordinate
${{{c}}}_{{{bc}}{,i}}$: Barrel coiling curvature at the i-th node
${{{c}}}_{{{i}}}$: Strip curvature at the i-th node
${{{c}}}_{0,{{i}}}$: Strip manufacturing curvature at the i-th node
${{{c}}}_{0}\left({{l}}\right)$: Strip manufacturing curvature as a function of the length curvature
$${{E}}$$: Young modulus
${{{E}}}_{{{desired}}}$: Elastic energy required to the spring
${{f}}\left(\mathbf{x}\right)$: Inverse problem’s objective function
${{{f}}}_{1}(\mathbf{x})$: First term of the inverse problem’s objective function
${{{f}}}_{2}(\mathbf{x})$: Second term of the inverse problem’s objective function
${{{f}}}_{3}(\mathbf{x})$: Third term of the inverse problem’s objective function
${{h}}\left({{l}}\right)$: Strip thickness as a function of the length coordinate
${{{h}}}_{{{i}}}$: Strip thickness at the i-th node
${{{h}}}_{1,{{max}}}$: Maximum admissible strip thickness at node one
${{{h}}}_{1,{{min}}}$: Minimum admissible strip thickness at node one
${{{h}}}_{{{m}},{{max}}}$: Maximum admissible strip thickness at the last node
${{{h}}}_{{{m}},{{min}}}$: Minimum admissible strip thickness at the last node
${{{K}}}_{{{penalty}}}$: Penalty constant for the nonlinear conditions that do not hold
${{{K}}}_{{{torque}}/{{mass}}}$: Weight of the torque curve with respect to the mass in the objective function
${{k}}$: Strip polar slope
${{{k}}}_{{{max}}}$: Maximum admissible strip polar slope
${{{k}}}_{{{min}}}$: Minimum admissible strip polar slope
$${{L}}$$: Strip length
${{{L}}}_{{{max}}}$: Maximum admissible strip length
${{{L}}}_{{{min}}}$: Minimum admissible strip length
${{l}}$: Strip length (axial) coordinate
${{N}}$: Number of arbor angle steps in the calculation of the torque curve
$${{{N}}}_{{{ind}}}$$: Population size
$${{M}}$$: Strip mass
${{{M}}}_{{{ideal}}}$: Ideal mass (theoretical minimal mass of a spring with the desired torque curve)
${{m}}$: Number of strip nodes
${{R}}\left({{l}}\right)$: Strip manufacturing radius as a function of the length curvature
$${{{r}}}_{{{H}}}$$: Barrel radius
${{{r}}}_{{{H}},{{max}}}$: Maximum admissible barrel radius
${{{r}}}_{{{H}},{{min}}}$: Minimum admissible barrel radius
$${{{r}}}_{{{S}}}$$: Arbor radius
${{{r}}}_{{{S}},{{shear}}}$: Barrel radius at which the material shear limit is reached
${{{r}}}_{{{S}},{{max}}}$: Maximum admissible arbor radius
${{{r}}}_{{{S}},{{min}}}$: Minimum admissible arbor radius
${{T}}\left(\Phi \right)$: Spring torque–angle curve
${{{T}}}_{{{desired}}}\left(\Phi \right)$: Desired torque-arbor angle curve
$${{V}}$$: Strip volume
$\mathbf{x}$: Vector of characteristics of a spiral spring
${{{x}}}_{{{ac}}{,i}}$: *x* Coordinate of the arbor-coiled strip at the i-th node
${{{x}}}_{{{bc}}, i}$: *x* Coordinate of the barrel-coiled strip at the i-th node
${{{y}}}_{{{ac}}, i}$: *y* Coordinate of the arbor-coiled strip at the i-th node
${{{y}}}_{{{bc}}, i}$: *y* Coordinate of the barrel-coiled strip at the i-th node

## Greek letters

$\mathrm{\Delta \Phi }$: Arbor rotation range of the spring
$\Delta {\Phi }_{{{desired}}}$: Desired arbor rotation range
$\Phi$: Arbor angular position
${\Phi }_{{{A}}}$: Angular position of the strip end attached to the arbor
${\Phi }_{{{B}}}$: Angular position of the strip end attached to the barrel
$\uprho$: Strip material density
${\uprho }_{{{E}}}$: Strip energy density
${\upsigma }_{{{adm}}}$: Elastic tensile limit
${\uptau }_{{{adm}}}$: Elastic shear limit

## Subscripts

$0$: Manufacturing (not assembled)
${\Phi }_{max}$: Maximum arbor angle reached by the spring
$$A$$: Arbor
$$ac$$: Arbor coiling
$$adm$$: Admissible
$$B$$: Barrel
$$bc$$: Barrel coiling
$$E$$: Energy
$$H$$: Barrel (housing)
$$ind$$: Individuals
$$max$$: Maximum
$$min$$: Minimum
$$S$$: Arbor (shaft)
