# Supplementary material for: Automated resolution of the spiral torsion spring inverse design problem
Source: Sci Rep. 2024 Feb 5;14:2956. doi: 10.1038/s41598-024-53404-6 (PMC10844218; doi:10.1038/s41598-024-53404-6)
Supplement: Supplementary file 1 — Supplementary Information. [file 41598_2024_53404_MOESM1_ESM.docx]

**AUTOMATED RESOLUTION OF THE SPIRAL TORSION SPRING INVERSE DESIGN PROBLEM**

Alejandro Silva^1, †^, Gonzalo López-Navarrete^2^, Carlos García-Martos^2^, Juan Manuel Muñoz-Guijosa^2, *, †^

**Supplementary information**

**List of symbols**

| $c_{0,i}$ | Strip manufacturing curvature at the i-th node |
| --- | --- |
| $c_{0}\left( l \right)$ | Manufacturing strip curvatures as a function of the length coordinate |
| $c_{ac,i}$ | Arbor coiling curvature at the i-th node |
| $c_{ac}\left( \Phi\right)$ | Arbor coiling curvature as a function of the coil angle coordinate |
| $c_{ac}\left( l \right)$ | Arbor coiling curvature as a function of the length coordinate |
| $c_{bc,i}$ | Barrel coiling curvature at the i-th node |
| $c_{bc}\left( \Phi\right)$ | Barrel coiling curvature as a function of the coil angle coordinate |
| $c_{i}$ | Strip curvature at the i-th node |
| $E_{p}$ | Total strip potential energy |
| $E_{pe}$ | Total strip elastic energy |
| $E_{pe,i}$ | Total strip elastic energy at the i-th node |
| $E_{pg}$ | Total strip gravitational energy |
| $E_{pg,i}$ | Total strip gravitational energy at the i-th node |
| $EI_{i}$ | Strip bearing stiffness of the i-th element |
| $EI\left( l \right)$ | Strip bearing stiffness as a function of the length coordinate |
| $g$ | Gravitational acceleration |
| $h_{i}$ | Strip thickness at the i-th node |
| $h\left( \Phi\right)$ | Strip thickness as a function of the coil angle coordinate |
| $r_{H}$ | Barrel radius |
| $r_{S}$ | Arbor radius |
| $R\left( \Phi\right)$ | Strip manufacturing radius as a function of the coil angle coordinate |
| $R\left( l \right)$ | Strip manufacturing radius as a function of the length curvature |
| $x_{i}$ | Strip x coordinate at the i-th node |
| $y_{0,i}$ | Strip y coordinate in the manufacturing position at the i-th node |
| $y_{i}$ | Strip y coordinate at the i-th node |

**Greek letters**

| $\Delta L_{i}$ | Length of the i-th element |
| --- | --- |
| $\Phi_{0}$ | Initial arbor rotation angle for the calculation of the spring torque curve |
| $\Phi_{N-1}$ | Final arbor rotation angle for the calculation of the spring torque curve |
| $\Phi_{max}$ | Desired arbor rotation range |
| $\Phi_{n}$ | Fixed n-th arbor rotation angle |
| $\Phi_{A}$ | Angular position of the strip end attached to the arbor |
| $\Phi_{B}$ | Angular position of the strip end attached to the barrel |
| $\Phi\left( l \right)$ | Arbor angular as a function of the length coordinate |
| $\varphi_{i}$ | Angle of the i-th element |
| $\mu_{i}$ | Mass per unit length of the i-th element |

**Subscripts**

| $0$ | Manufacturing (not assembled) |
| --- | --- |
| $ac$ | Arbor coiling |
| $bc$ | Barrel coiling |
| $H$ | Barrel (housing) |
| $max$ | Maximum |
| $n$ | Arbor angle step for the calculation of the spring torque curve |
| $p$ | Potential |
| $pe$ | Potential elastic |
| $pg$ | Potential gravitational |
| $S$ | Arbor (shaft) |

### S1 The direct problem of the spiral spring: calculation of the torque-arbor rotation curve

This section summarizes the formulation for the resolution of the spiral spring's direct problem presented by [27], which is the core of the methodology for the automated design of spiral springs proposed in this manuscript.

In line with other spiral spring models found in the Literature for the calculation of their mechanical properties, the five elementary assumptions for the modelling of the strip are:

1. Monolithicity: strip deformation can be modeled by means of the Bernoulli beam model. For each element, a different cross-section geometry can be defined. This is how springs with variable cross-section can be defined.
2. Inextensibility: the axial deformation is negligible with respect to that caused by bending.
3. Proper operation: there is no strip self-interference during the whole spring operating range, as expected in any correctly designed and operated spiral spring.
4. No strip out-of-plane deflection nor torsion deformation: strip deformation occurs only in the spring plane.
5. Finally, we assume that the spring will be operated in static or quasi-static operation.

These assumptions are maintained for the resolution of the inverse design problem of the spiral spring. They enable simplifying the coil modelling while guaranteeing an accurate simulation. Each of them has been properly justified in [27].

A spring is defined by its barrel radius $r_{H}$, arbor radius $r_{S}$, strip length $L$ and a manufacturing curvature $c_{0}\left( l \right)$ that varies along the strip length, measured from its end attached to the barrel (Fig. 1). The spring lies on the $xy$ Cartesian plane, and the $z$ axis of the global reference frame is coincident with the arbor rotation axis. The clamping between the strip and the barrel is located at an angle $\Phi_{B}$ with respect to the $x$ axis. The strip bending stiffness $EI\left( l \right)$ can vary along the strip length by changing the geometry of its cross-section and/or the strip material properties.

The strip is split into $m$ nodes and $m-1$ elements with identical length (Fig. S1.1). Each element $i$ is located between nodes $i$ and $i+1$, has a radial thickness $h_{i}$ and a mass per unit length $\mu_{i}$. The coordinates of each node are $x_{i}$ and $y_{i}$. The first ($i=1$) and last ($i=m$) nodes coincide with the strip attachments to the barrel and the arbor, respectively.

Given the curvatures $\left\{ c_{2},\text{...},c_{m-1} \right\}$ of the strip nodes, the arbor angular position $\Phi_{A}$ and the coordinates of the nodes can be calculated with the iterative application of Eq. S1.1 to S1.7:

$\varphi_{1}=\Phi_{B}+\pi/2,$ (S1.1)

$\varphi_{i}=\varphi_{i-1}+c_{i}\Delta L_{i-1},\quad i=2,\text{...},m-1,$ (S1.2)

$\Phi_{A}=\varphi_{m-1}-\pi/2,$ (S1.3)

$x_{1}=\left( r_{H}-h_{1}/2 \right)\text{cos}\left( \Phi_{B} \right),$ (S1.4)

$y_{1}=\left( r_{H}-h_{1}/2 \right)\text{sin}\left( \Phi_{B} \right),$ (S1.5)

$x_{i}=x_{i-1}+\Delta L_{i-1}\text{cos}\left( \varphi_{i-1} \right),\quad i=2,\text{...},m,$ (S1.6)

$y_{i}=y_{i-1}+\Delta L_{i-1}\text{sin}\left( \varphi_{i-1} \right),\quad i=2,\text{...},m.$ (S1.7)


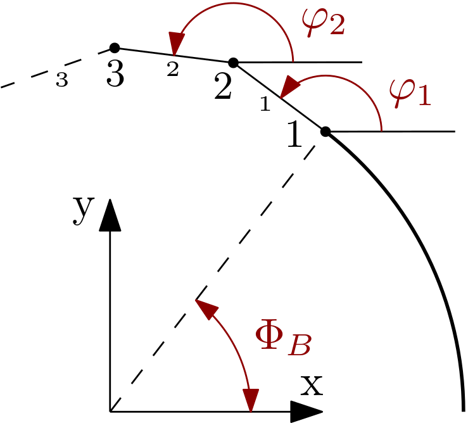


**Fig. S1.1** Node and element indices, element and position angles of strip coupling to barrel. From [27].

If dissipation is neglected, the potential energy of the spiral spring can be expressed as the sum of its elastic ($E_{pe}$) and gravitational potential energies ($E_{pg}$),

$E_{p}=E_{pe}+E_{pg}=\sum_{i=1}^{m-1} E_{pe,i}+\sum_{i=1}^{m-1} E_{pg,i}.$ (S1.8)

The elastic and gravitational potential energies of each element can be expressed as

$E_{pe,i}=\frac{1}{2}EI_{i}\Delta L_{i}\left( c_{i}-c_{0,i} \right),\quad E_{pg,i}=\mu_{i}g\Delta L_{i}\left( y_{i}-y_{0,i} \right),$ (S1.9)

where $E$ and $I_{i}$ are the material Young’s modulus and the cross-section moment of inertia of the i-th element, respectively, and $\Delta L_{i}$ is the i-th element length. We assume a constant Young's modulus $E$ along the strip length.

The first step of the algorithm is to calculate the curvatures that the strip adopts in its mounting position under zero external torque. These can be obtained by solving the following nonlinear restrained optimization problem expressed by an objective function and a set of nonlinear constraints. The aim is to find a set of strip curvatures $c_{2},\text{...},c_{m-1}$ that minimize the strip's potential energy:

$\min_{c_{2}\text{...}c_{m-1}} E_{p},$ (S1.10)

$c_{bc,i}\leq c_{i}\leq c_{ac,i},\quad i=2,\text{...},m-1,$ (S1.11)

$\sqrt{x_{m}^{2}+y_{m}^{2}}-\left( r_{S}+\frac{h_{m}}{2} \right)=0,$ (S1.12)

$\left( \Phi_{A} \right)_{-\pi,\pi}-\text{arctan}\left( y_{m},x_{m} \right)-k=\text{0.}$ (S1.13)

The constraint in Eq. S1.11 guarantee that the curvatures are bounded between the coiling curvatures around the barrel, $c_{bc}\left( l \right)$, and the arbor, $c_{ac}\left( l \right)$. The constraints in Eq. S1.12 and S1.13 guarantee that the strip is attached to the arbor with a fixed clamping angle $k$. The calculation of the coiling curvatures $c_{i,bc}$ and $c_{i,bc}$ for a given spring geometry is described in Appendix One of [27]. It the coil has variable thickness; the calculation of the coiling curvatures becomes more complex and requires solving a constrained nonlinear equation. A detailed description of this calculation is found below in Section 3 of this supplementary information document.

After calculating the mounting position, a similar nonlinear optimization problem can be formulated to obtain the torque-angle curve of the spring. Given a set of arbor rotation angles $\Phi_{n}$ ($n=0,\text{...},N-1$), where $\Phi_{0}$ coincides with the arbor mounting angle $\Phi_{A}$, and $\Phi_{N-1}$ equals the maximum arbor rotation angle $\Phi_{max}$, the arbor torque that realizes each rotation angle can be obtained by finding the set of strip curvatures that minimize the coil potential energy $E_{p}$ as in Eq. S1.10 with constraints S1.11 and S1.12, and substituting the constraint Eq. S1.13 by the following equation, which forces the arbor to rotate the desired angle,

$\left( \Phi_{n} \right)_{-\pi,\pi}-\text{arctan}\left( y_{m},x_{m} \right)-k=\text{0.}$ (S1.14)

The result of this optimization problem is an arbor angle-potential energy curve $E_{p}\left( n \right)=E_{p}\left( \Phi_{n} \right)$. This curve can be smoothed by fitting the potential energies to a sixth-degree polynomial (or of lesser degree if it is overfitted), obtaining $E_{p}\left( \Phi\right)$. Differentiation of this polynomial gives the desired torque-angle curve $T\left( \Phi\right)$,

$T\left( \Phi\right)=\frac{dE_{p}\left( \Phi\right)}{d\Phi}.$ (S1.15)

This function is the main algorithmic output of the direct problem. As a by-product, the algorithm also gives the coil’s quasi-static length-curvature functions $c\left( l \right)$ corresponding to arbor rotation angles ${\Phi_{1},\ldots,\Phi}_{n}$. Spring elasticae can be obtained from $c\left( l \right)$ by applying iteratively Eq. S1.1 to S1.7.

Finally, we note that this algorithm can be sped up considerably by applying polynomial interpolation to the set of strip curvatures $\left\{ c_{2},\text{...},c_{m} \right\}$. By doing so, the number of variables to optimize in Eq. S1.10 drops from $m$ to the number of parameters of the approximating polynomials. If such a number is well chosen, the loss in calculation accuracy is negligible. In this work, the modified Akima cubic Hermite interpolating function [39] has been employed to approximate the strip length-curvature function of the spiral springs.

**
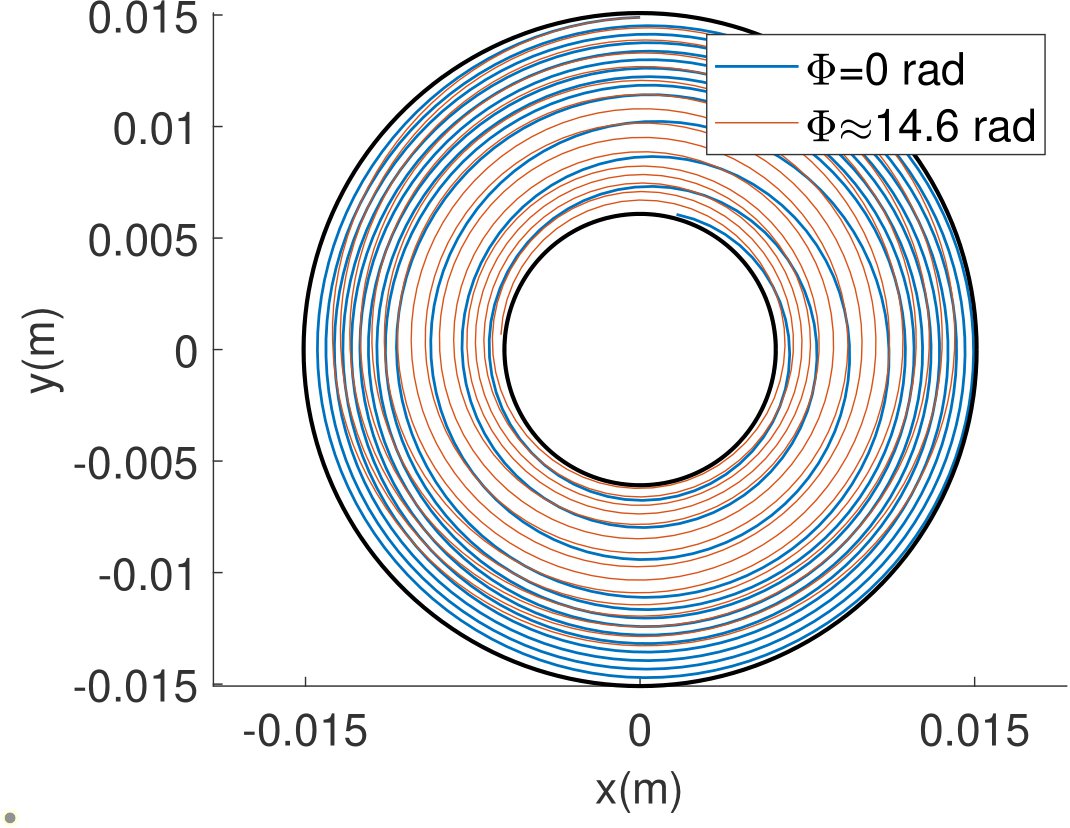
**This formulation was put to the test on the spring No.4 of Queener et al [25]. The experimental results shown in this publication were used in [27] as validation of the direct problem outputs. Two spring elasticae obtained minimizing Eq. S1.14 and then applying Eq. S1.1 to S1.13 are shown in Fig. S1.2**(a)**. The elasticae correspond to the arbor resting position (at $\Phi=0$) and to a forced shaft rotation of 14.2 rad. Fig. S1.2**(b)** and 3**(c)** show, respectively, the potential energy obtained for different arbor rotation angles and the calculated arbor torque at each rotation angle. Fig. S1.2(c) also shows the coiling (red) and uncoiling (blue) torque curves, which consider the hysteresis caused by friction between spires in contact. [27] further describes these results and the validation and shows how to introduce friction in the calculation of the torque-angle curves.**
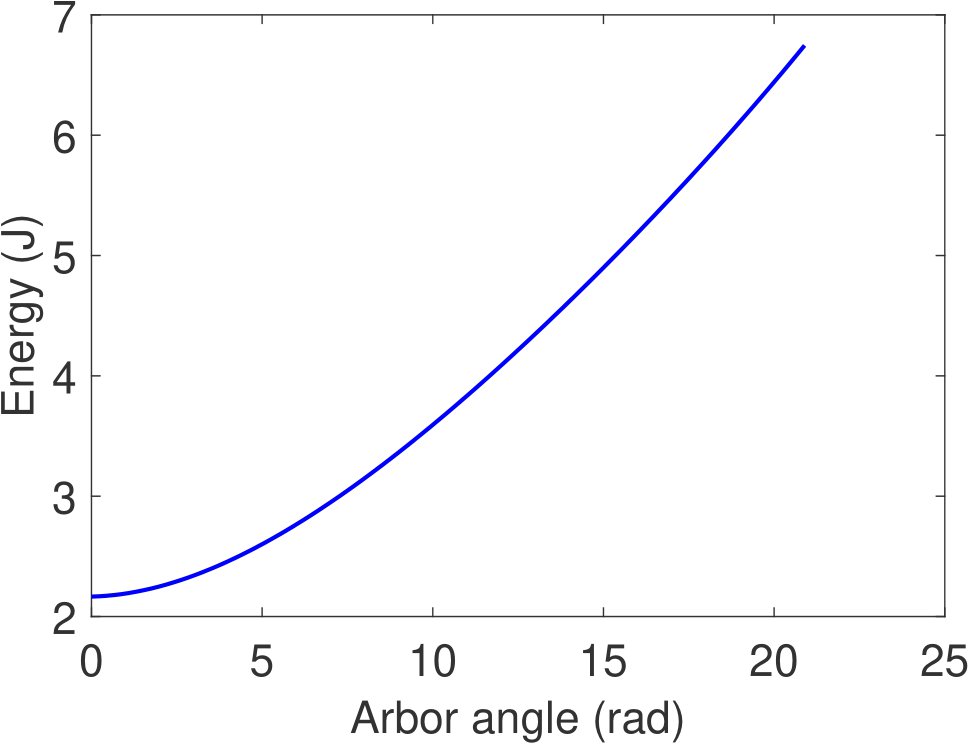
(a)** **(b)**

**(c)**

**Fig. S1.2** Elasticae, potential energy–angle curve and torque–angle curve of the Queener \& Wood spring as the solutions of the energy minimization problem (Eq. S1.10). **(a)** Mounting (0 rad) and loaded (~14.6 rad) spring elasticae. **(b)** Total potential energy. **(c)** Spring theoretical torque curves, and in black, the experimental torque curves of the spring No. 4 of Queener et al [25]. From [27].

### S2 The genetic algorithm for the resolution of the inverse spring design problem

The inverse problem algorithm follows this iterative procedure to find an optimal solution (Figure **S2.1**): first, a candidate solution vector$x$ is generated, then the direct problem (Section 2) is solved on that spring which is uniquely defined by the proposed vector of characteristics $\boldsymbol{x}$ to calculate its torque-angle curve, $T\left( \Phi\right)$. Then, the objective function $f\left( \boldsymbol{x} \right)$ (Eq. 26 of the manuscript) is evaluated at $\boldsymbol{x}$ with the computed torque-angle curve. To evaluate this objective function, the torque curve $T\left( \Phi\right)$ is compared with the desired torque-angle curve, $T\left( \Phi\right)$; the nonlinear constraints are checked on $\boldsymbol{x}$, and the mass of spring $\boldsymbol{x}$, $M$, is calculated. If the value of the objective function $f\left( x \right)$ is not a minimum in the domain of feasible solutions, then $\boldsymbol{x}$ is ruled out as the solution of the optimization problem and a new candidate solution $\boldsymbol{x}$ is generated and evaluated. This process is repeated until an optimal solution is found.

In a genetic algorithm, an initial population of individuals $\left\{ \boldsymbol{x}_{1},\text{...},\boldsymbol{x}_{N} \right\}$ (i.e., of candidate springs with vectors of characteristics such as Eq. S1.16 in the set of nine-tuples of real numbers) is randomized at the beginning. This randomization must ensure that all the individuals of the initial population hold all the linear constraints (Eq. 6 to 14 of the manuscript). To initialize this set, we follow these steps:

1. Randomize $N_{ind}$ individuals.
2.
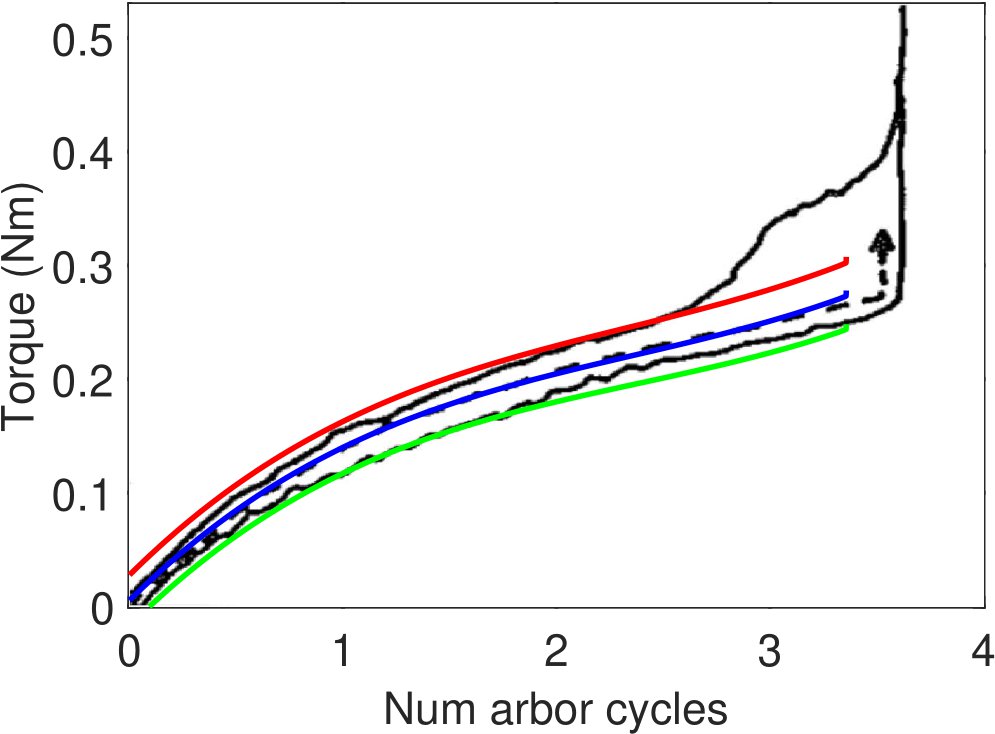
Set $i=1$.
3. While $i<N_{ind}$,
   1. randomize a vector of characteristics $\boldsymbol{x}_{i}$, considering, for each feature contained in the vector, a homogeneous probability distribution between the upper and lower bounds of the linear constraints (Eq. 6 to 14 of the manuscript).
   2. add one to $i$,
   3. re-iterate.

Then, Eq. 41 is evaluated at each $\boldsymbol{x}_{i}$, $i=1,\text{...},N_{ind}$. The lower the value of the objective function $f\left( \boldsymbol{x}_{i} \right)$, the higher the fitness of the spiral spring represented by $\boldsymbol{x}_{i}$, and therefore, the higher the probability that that individual is selected for survival and interbreeding (crossover) with other surviving spiral springs, while randomly mutating the characteristics of their offspring. Both crossover and mutation should ensure that each individual’s features in the offspring are within the boundaries imposed by the linear constraints (Eq. 6 to 14 of the manuscript). After crossover and mutation, a new generation of $N_{ind}$ individuals holding the linear constraints is established. The evaluation, selection and interbreeding process is iterated until the whole population converges to an optimal solution of the inverse design problem. If the number of individuals is large enough and with the proper optimization parameters, convergence to a global minimum can be guaranteed [38].


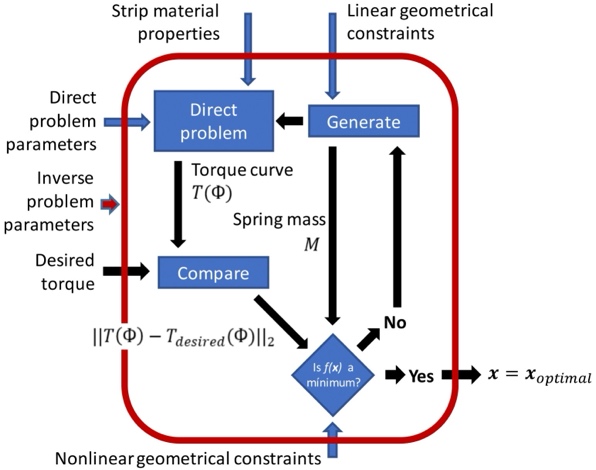


**Fig. S2.1** The strategy followed for the resolution of the inverse design problems of spiral springs.

### S3 calculation of the coiling curvatures of a strip with variable thickness

The objective is to calculate the strip coiling curvatures around the barrel, $\left\{ c_{bc\text{,1}},\text{...},c_{bc,m} \right\}$, and the arbor, $\left\{ c_{ac\text{,1}},\text{...},c_{ac,m} \right\}$, given the barrel and arbor radii, $r_{H}$ and $r_{S}$, and the strip element lengths and thicknesses, $\Delta L_{i}$ and $h_{i}$ for all $i=1$ to $m-1$, considering the strip thickness at the m-th node (denoted $h_{m}$) equal to the thickness of the element $m-1$.

When the strip is coiled either on the barrel surface or around the arbor, its geometry holds these characteristics:

1. for the barrel-coiled spring, the curvature of the outer strip surface at the first node ($l=0$) equals $1/r_{H}$; and for the arbor-coiled spring, the curvature of the inner strip surface at the last node ($l=L$) equals $1/r_{S}$,
2. the strip coils are wrapped and in full contact with one another, and
3. the strip curvature varies smoothly and monotonously with its length.

To simplify the mathematical formulation of these three conditions and of the resolution of the coiling problem, we can consider the coiling curvatures and the thicknesses as functions of the coil strip length coordinate $l$, denoting them as $c_{bc}\left( l \right)$, $c_{ac}\left( l \right)$ and $h\left( l \right)$. Since there is a one-to-one relation between the length coordinate$l$ of a strip node and its absolute angle $\Phi$ with respect to the x Cartesian axis ($\Phi\left( 0 \right)=\Phi_{B}$) we can also write the coiling curvatures and the thicknesses as functions of the angular coordinate $\Phi$: $c_{bc}\left( \Phi\right)$, $c_{ac}\left( \Phi\right)$ and $h\left( \Phi\right)$.

With this notation, the first characteristic can be mathematically expressed as $c_{bc}\left( 0 \right)=1/\left( r_{H}-h\left( 0 \right)/2 \right)$ for the coiling around the barrel, and $c_{bc}\left( L \right)=1/\left( r_{S}+h\left( L \right)/2 \right)$ for the coiling around the arbor. The second characteristic can be formulated as follows: select a node along the strip with axial coordinate $l=l_{1}$ and absolute angle $\Phi_{1}=\Phi\left( l_{1} \right)$; then, take its two adjacent nodes, those whose absolute angles are $\Phi_{2}=\Phi_{1}+2\pi$ and $\Phi_{3}=\Phi_{1}-2\pi$. If, as in most operational spiral springs, the strip thickness along its length is much smaller than the distance from the strip nodes to the arbor rotation axis (simply called hereafter the *radius* of a strip node and denoted as $R\left( l \right)$), we can assume within tolerance that

$R\left( \Phi_{1} \right)-\frac{h\left( \Phi_{1} \right)}{2}=R\left( \Phi_{2} \right)+\frac{h\left( \Phi_{2} \right)}{2},$ (S3.1)

$R\left( \Phi_{1} \right)+\frac{h\left( \Phi_{1} \right)}{2}=R\left( \Phi_{3} \right)-\frac{h\left( \Phi_{3} \right)}{2},$ (S3.2)

$c\left( l \right)=\frac{1}{R\left( l \right)},$ (S3.3)

for all $l\in\left[ 0,L \right]$.

Finally, a function $R\left( l \right)$ must be found holding $c_{bc}\left( 0 \right)$ or $c_{bc}\left( L \right)$ and Eq. S3.3 so that it represents accurately the elastica of a coil strip. Particularly, we obtained very good results by approximating $R\left( l \right)$ with a piecewise cubic Hermite interpolating polynomials with four equidistant sample nodes: the first and last strip nodes and two intermediate nodes.

With the aforementioned considerations, if we hypothesize that $h\left( l \right)<<r\left( l \right)$ for all $l\in\left[ 0,L \right]$ whether the strip is coiled around the arbor or on the barrel, the coiling curvature functions $c_{bc}\left( l \right)$ and $c_{ac}\left( l \right)$ can be estimated by solving the following problem: find a function $r\left( \Phi\right)$ so that this $L^{2}$ norm equals zero in the interval $\Phi\in\left[ \Phi_{B},\Phi\left( L \right)-2\pi\right]$:

$\left\| R\left( \Phi\right)-\frac{h\left( \Phi\right)}{2}-\left( R\left( \Phi+2\pi\right)+\frac{h\left( \Phi+2\pi\right)}{2} \right) \right\|_{2}=0,$ (S3.4)

under the constraint $r\left( \Phi_{B} \right)=r\left( 0 \right)=r_{H}-h\left( 0 \right)/2$ for barrel coiling, or $R\left( L \right)=r_{S}-h\left( L \right)/2$ for arbor coiling.

Then, the strip coiling curvatures can be calculated as the inverses of the coiling radii: $c\left( l \right)=1/R\left( l \right)$ for all $l\in\left[ 0,L \right]$. Note that since we use interpolating polynomials with four sample nodes, the equation above has only four unknowns.
